# Creating a Collaborative Healthcare Environment: Early Exposure to Multidisciplinary Roles and Dynamics Through an Interprofessional Seminar Series for Undergraduate Healthcare Students

**DOI:** 10.7759/cureus.83664

**Published:** 2025-05-07

**Authors:** Siena Blackwell, Aaron Pathak, Brianna Etoria, Peter Boedeker

**Affiliations:** 1 School of Medicine, Baylor College of Medicine, Houston, USA; 2 College of Education, University of Houston, Houston, USA; 3 Department of Education, Innovation, and Technology, Baylor College of Medicine, Houston, USA

**Keywords:** collaboration, crna, education, interdisciplinary, interprofessional, medical education, orthotics and prosthetics, physician assistant, srna

## Abstract

Introduction

Interprofessional collaboration and communication among members of the healthcare team are fundamental in providing high-quality patient care. Students at healthcare education institutions are in a unique position to develop their understanding of the patient care team before entering the workplace. However, education among these different programs remains largely segregated. A lack of interprofessional education (IPE) is a missed opportunity to educate students on the functions of other professions and how to collaborate and communicate effectively within an interdisciplinary team. To address these gaps, a student-led team piloted a seminar series involving students and faculty of various health professions.

Methods

This study was conducted at a private health sciences university in Houston, Texas. Programs offered at this university included the following: doctor of medicine (MD), physician assistant (PA), certified registered nurse anesthetist (CRNA), genetic counseling, and orthotics and prosthetics (O&P). The seminar series included four optional one-hour to one-and-a-half-hour sessions consisting of three panel discussions and one small group session. Sessions featured speakers representing each of the participating programs and were guided by prepared questions to address the topic of the session, along with allotted time for student questions. The three panel session topics were as follows: The History and Development of the Interdisciplinary Team, Scope of Practice Panel Discussion, and A Day in the Life of the Professional. The small group session featured the following topic: Interdisciplinary Collaboration and Putting a Stop to Bullying. An optional survey was administered following each session. The Wilcoxon paired rank-sum test was used to compare responses on the retrospective survey items, while qualitative responses were analyzed using inductive coding methods, allowing patterns to be identified without predetermined categories.

Results

Eighty-five survey responses were received over the entire series. Quantitative analysis indicated significant increases in the self-reported understanding of the key measurement for each session (p<0.001-0.02) and improvement in their interdisciplinary communication skills (p<0.001). Qualitative feedback largely focused on an appreciation for the question-and-answer format of the sessions and the quality of the speakers.

Conclusions

This seminar series provided a valuable opportunity for students in different healthcare programs to gain exposure to realistic scenarios and actual experiences from panelists and learn about the roles of their future colleagues. Analysis showed significant increases in the perceived understanding of the other health professions following the series. Future endeavors include recreating the seminar series at this institution to gather additional data, studying past participants in the clinical setting to assess the practical impact of experiences gained through these sessions, and helping other institutions create similar educational programs.

## Introduction

The healthcare team functions optimally when the shared expertise among professionals from a multitude of unique specialties is effectively integrated to support patient care. Interdisciplinary collaboration has been shown to have positive effects on patient outcomes while improving job satisfaction among healthcare professionals [1,2]. However, the educational background, responsibilities, and scope of practice greatly vary among healthcare professions; this reality leaves students and professionals prone to misconceptions about the contribution that each member of the care team can make [3,4]. During interprofessional education (IPE), students gain early exposure to the variety of healthcare professions that they will interact with when providing patient care in an effort to enhance the learners’ understanding of, and ability to collaborate on, a professionally diverse team [5,6].

The need for IPE

The lack of exposure to IPE exists across professions and has implications for team cohesion. Undergraduate medical, nursing, and physician assistant (PA) students have heterogenous understandings of professions outside of their area of study [7]. Little information can be found regarding perceptions of certified prosthetics and orthotics (CPO) and certified genetic counselor (CGC) students on the interdisciplinary team, likely due to the relative novelty of these fields, with their first degree programs originating in the 1960s [8,9]. Concerningly, mistreatment and bullying have been linked to limited exposure to IPE [10-12]. To address the lack of interprofessional understanding and avoid instances of bullying that may result, a variety of IPE programs have been developed.

Elements of effective IPE

IPE has been implemented in a variety of ways, ranging from half-day workshops to longitudinal programs implemented over a year or more [13]. Guidance on the implementation of effective IPE includes allowing discussion time, peer-to-peer learning opportunities, and education on the various roles and responsibilities of the members of the healthcare team [14]. Guraya and Barr found a “significant impact of IPE educational intervention on students’ knowledge, skills, and attitudes” across undergraduate medical years with a notable benefit when incorporating clinicians who can help students understand the practical application of IPE [3]. Studies have also demonstrated improved outcomes in student satisfaction with small group formats compared to lectures [15]. In sum, effective IPE has been shown to include discussion and peer-to-peer learning in small groups with involvement by clinicians sharing their lived experience.

Addressing the challenges

A pilot seminar series was developed by students to address the lack of knowledge of other professions on the healthcare team, with consideration of best practice recommendations for implementing IPE. Each session in this study was grounded in a conceptual framework focused on understanding the roles of members of the healthcare team, interprofessional communication strategies, and bullying mitigation as a healthcare profession student, utilizing theory from Averbuch et al. [15] and intervention design from Berger et al. [16]. Experienced clinicians from various specialties were invited to speak at each session to share their perspective on the application of IPE in the professional setting. Learners were provided ample time for the discussion and sharing of their experiences. The overarching objective of the series was to enhance students’ understanding of other professionals’ backgrounds, training, and scopes of practice while fostering respectful and professional collaboration. The series therefore focused on specific elements of knowledge gain and confidence with navigating the interprofessional team. Here, we present the evaluation of the seminar series.

## Materials and methods

Site and participants

This study was conducted at a single institution in Houston, Texas, that offers multiple healthcare profession programs. Programs offered include the following: doctor of medicine (MD), PA, certified registered nurse anesthetist (CRNA), CGC, and orthotics and prosthetics (O&P). Students in these programs were recruited to attend four panel sessions with different speakers representing each of the participating programs. Sessions were advertised using physical flyers around the campus and email distribution. Student participation was voluntary, and attendance at these sessions did not affect their grades or coursework in any way. Students could join any number of sessions but were not required to attend any session as part of their program. Attendees were invited to voluntarily complete a survey following each session that they attended. This study was approved by the Institutional Review Board of Baylor College of Medicine (BCM) and Affiliated Hospitals under protocol H-54123.

A total of 85 survey responses were included in our study over the four sessions. Some responses may come from the same individuals attending more than one session, given the anonymous nature of the survey. Session 2 (scope of practice) had the largest sample size at 26 students, while session 4 (role of professions) had the least at 14 students. The majority of characteristics of the participants included 64 (75.29%) women, 40 (47.06%) medical doctorate program students, and 49 (51.58%) who self-identified as non-Hispanic White respondents (Table 1).

**Table 1:** Combined Demographics Across Four Sessions MD/PhD, doctor of medicine/doctor of philosophy; CGC, certified genetic counselor; DNP-CRNA, doctor of nursing practice-certified registered nurse anesthetist; PA, physician assistant; O&P, orthotics and prosthetics

Characteristics	Frequency (%)
Gender	
Male	21 (24.71)
Female	64 (75.29)
Enrolled program	
Medical student	40 (47.06)
MD/PhD	8 (9.41)
CGC	6 (7.06)
DNP-CRNA	1 (1.18)
PA	20 (23.53)
O&P	10 (11.76)
Race	
Non-Hispanic White	49 (51.58)
Asian	34 (35.79)
Hispanic	9 (9.47)
Black	2 (2.11)
Prefer not to answer	1 (1.05)

Workshop session design

The four one-hour sessions were designed by a team of education professionals and students from various programs at BCM and spaced over a three-month period (session objectives in Table 2). Each session targeted objectives that were designed around identified problems in interprofessional education. Broadly, the objectives of the sessions were to introduce students to other healthcare professions, facilitate the discussion of interprofessional communication strategies, and provide opportunities to learn about bullying mitigation through role-play scenarios. Bullying mitigation was addressed as a common theme in each session through shared experiences by panelists and the discussion of the importance of reporting and how and when to make a report. Panel discussions were guided by prepared questions to address the topic of the session; however, students were encouraged to ask questions throughout each session (Table 2). Panelists were composed of faculty members and did not include trainees.

**Table 2:** Session Objectives N/A: not available

Session	Objectives	Guided Question
One: history and development of the interdisciplinary team	Understand the educational and professional requirements of each profession	What is your educational background, and what certifications do you hold?
	Learn the history of each profession and its development to modern-day practice	N/A
Two: scope of practice panel discussion	Gain perspective of different professionals on the scope of practice of their represented profession	What is your scope of practice, and how does it impact how you work together on an interprofessional team?
	Facilitate interdisciplinary discussion through audience questions directed to the panel	N/A
	Demonstrate how scope differs among professions and the strengths and limitations of what each profession can offer to the patient care team	In what ways do you believe your scope is appropriate, and what, if any, are areas your scope can be changed or improved?
Three: interdisciplinary collaboration and putting a stop to bullying	Participate in small group discussions on interprofessional conflict and bullying	N/A
	Discuss methods to navigate discord in the workplace	N/A
	Learn strategies for effective communication and collaboration on an interprofessional team	N/A
Four: a day in the life of the professional	Gain understanding of the daily workflow and common workplace challenges for each profession	What does an average day look like for you, including daily tasks, patient interactions, and interdisciplinary collaboration?
	Demonstrate common instances of interdisciplinary collaboration	What are examples of how you work together with other professions?
	Panelists discuss the reasons that they chose their profession and the components of their career that they find enjoyable and challenging	How did you choose your profession? What parts of your career do you find enjoyable, and what parts are more challenging?

Session 1: History and Development of the Interdisciplinary Team

This session began with a brief lecture explaining how each profession evolved from its inception to modern times. It was followed by a panel discussion composed of professionals, including a PA, CRNA, CGC, and CPO. Panel discussion points were designed to promote discussion of how panelists have seen their profession change over the time they have been practicing. Following the guided discussion points, questions were invited from audience members who consisted of students from various programs.

Session 2: Scope of Practice Panel Discussion

This session focused on the legal and practical scope of practice in each profession. A similar format as session 1 was utilized with a lecture to introduce the topic, followed by a panel discussion featuring a PA, CRNA, CGC, CPO, and an MD representative. Guided discussion topics included the role of advanced practice providers from their lens, as well as physician input. Panelists also discussed prescription capabilities regarding controlled substances, treatments, and controlled medications. Audience members were again encouraged to ask questions following guided discussion points.

Session 3: Interdisciplinary Collaboration and Putting a Stop to Bullying

This session was uniquely formatted to include a brief lecture describing the statistics of bullying in healthcare, followed by small group discussions. The small groups were formatted to include hypothetical scenarios of interprofessional conflict to expose students to examples of workplace bullying. Students were encouraged to share their perspectives on how they perceived each scenario and what they would do if they encountered a similar instance. The small group sessions ended with a discussion regarding how professionals can support each other in the workplace regarding consultations, rotations, and everyday tasks that come with working together.

Session 4: A Day in the Life of the Professional

The final session was composed of a guided panel discussion on an average day in the life of various professionals. The panelists described their workflow, common workplace challenges they encounter, and how they collaborate with other professions throughout their day. Panelists were also asked to explain the reasons why they chose their profession and the components of their career that they find enjoyable and challenging.

Data collection

Surveys were designed utilizing items with a five-point Likert scale to evaluate the effectiveness of each session using the first two levels of Kirkpatrick’s four levels of evaluation [17]. Level 1 questions addressed the participants’ perception of speakers’ knowledge, effectiveness of session format, and valuableness of attending the session. Level 2 questions evaluated the participants’ perceived confidence levels related to session objectives before and after attending the session. A retrospective, pre/post-survey format was used in which each participant was asked in a single survey to evaluate their confidence in the ability to complete a task or navigate a scenario prior to the session and after the session. The retrospective, pre/post-survey format was selected to ensure that self-evaluations of pre- and post-confidence levels were based on the same level of knowledge of the topic. An optional free-text response allowed participants to share why they did or did not think the format of the session was effective. Two additional optional free-text responses were included at the end of each survey: one requesting feedback for improvement and the other requesting any additional feedback. The participants were invited to complete each survey at the conclusion of each session.

Statistical analysis

Descriptive statistics were used to display combined characteristics of the participants and participant evaluation of session value. Participant feedback gathered through the session survey was included in this study. Some attendees may be represented more than once if they came to multiple sessions, since no identifiers were kept. The Wilcoxon paired rank-sum test was used to compare responses on the retrospective survey items. Questions that were present in all four session surveys were used for data analysis. R version 4.3.1 (R Foundation for Statistical Computing, Vienna, Austria) was used for statistical analysis. Qualitative data gathered from free-text responses were first read through multiple times to gain a comprehensive understanding of the content. An inductive coding approach was used: initial open coding was conducted line by line to capture key concepts without imposing predetermined categories. A preliminary set of codes was developed and iteratively refined as patterns and new insights emerged. After coding all responses, related codes were clustered together to develop overarching themes that reflected the participants’ experiences and perspectives. After one researcher completed the initial analysis, the coding procedures and findings were collectively reviewed and validated by the research team to strengthen the reliability and trustworthiness of the results.

## Results

Knowledge and confidence (Likert scale)

Revealed in the quantitative analysis of the survey data, the median student perception of each session’s value was 5 (IQR: 5-5) for all sessions (Table 3). Students’ self-reported understanding of each session’s objectives significantly increased following the attendance of each of the sessions (Table 4). Likewise, students’ self-reported confidence in their ability to collaborate with health professionals outside of their field of study significantly increased after each of the sessions (Table 5). Survey responses indicate that students found the sessions to be highly valuable and informative and to increase participant confidence in their interprofessional skills.

**Table 3:** Student Perception of Seminar Series Value

Session	Sample Size (n)	Median (IQR)
Session 1	25	5 (5-5)
Session 2	26	5 (5-5)
Session 3	20	5 (5-5)
Session 4	14	5 (5-5)

**Table 4:** Student Understanding of Key Measurement (Pre-Post Comparison) A value of V=0 indicates that the mean rank of every post-question is greater than the pre-question MD, doctor of medicine; CRNA, certified registered nurse anesthetist

Session	Key Measurement (Question Asked)	Sample Size (n)	Pre: Mean (SD)	Post: Mean (SD)	Wilcoxon	P-value
Session 1	My understanding of the basic history of medical education and basic responsibilities of MDs, PAs, CRNAs, genetic counselors, and orthotists and prosthetists	25	2.84 (0.98)	4.28 (0.68)	V=0	<0.001
Session 2	My understanding of the components of the scope of practice for health professionals outside of my field of study	26	2.81 (0.80)	4.27 (0.83)	V=0	<0.001
Session 3	My understanding of how to implement communication skills and professionalism during encounters of bullying that I may experience	20	3.10 (0.64)	4.25 (0.72)	V=0	<0.001
Session 4	My understanding of the role healthcare professionals in and outside of my field of study play within the whole of the healthcare team	14	3.29 (0.91)	4.50 (0.65)	V=0	0.002

**Table 5:** Student Confidence in Collaborating Outside of the Field of Study (Pre-Post Comparison) Question evaluated at the end of each session: “my confidence in collaborating with health professionals outside of my field of study.” A value of V=0 indicates that the mean rank of every post-question is greater than the pre-question

Session	Sample Size (n)	Pre: Mean (SD)	Post: Mean (SD)	Wilcoxon	P-value
Session 1	25	3.32 (0.95)	4.32 (0.69)	V=0	<0.001
Session 2	26	3.42 (0.90)	4.35 (0.69)	V=0	<0.001
Session 3	20	3.25 (0.91)	4.40 (0.68)	V=0	<0.001
Session 4	14	3.57 (0.51)	4.43 (0.65)	V=0	0.004
Combined	85	3.38 (0.86)	4.36 (0.67)	V=0	<0.001

Written feedback (free-text response)

The qualitative data collected through free-text response questions on the session surveys provided participants an opportunity to share open-ended feedback through three questions. The participants were asked whether they felt the format of delivery was logical and effective, what changes they would make to improve the quality of each talk, and for additional feedback, if any.

Across all four surveys, 21 participants responded to the question regarding session format. As to why the participants did or did not find the sessions logical and effective, seven (33.3%) found the sessions to be informative, and six (28.6%) described them as organized. In the first session survey, one respondent reported, “I liked how everything was laid out and the extra time for Q&A.” Reflecting on the third session, one respondent reported that their “small group speaker was very empathetic and knowledgeable.” Significantly, five (23.8%) respondents also left feedback about disorganization. When further investigated, four of five of these responses were related to session 2. This feedback will be used to inform the restructuring of this session in future implementations of the series.

Thirty-three responses were collected with feedback regarding changes to make to improve the quality of each talk. Twelve (36.4%) responses were either “N/A,” “none,” or compliments on the quality with no recommendations for changes. Ten (30.3%) responses were related to recommending changes to activities. Eight (24.2%) responses were recommendations for changes to activity structure. The most common ideas for structure changes were for group activities to be facilitated in smaller groups and better management of topic adherence regarding the questions and answers with panelists. Six (18.2%) respondents were interested in further increasing their opportunities for interprofessional collaborations. For example, one participant’s response after session 2 was as follows: “I would love to participate in longer interprofessional meetings.”

Across all session surveys, only two comments were made in response to the request for additional feedback; therefore, there are no findings to report. Overall, the respondents appreciated the knowledge gained during the series and were mostly pleased with the organization of the sessions, apart from session 2. Most commonly, the participants did not have feedback on changes they would make to improve the quality of the series. However, ideas on improving session activities will be considered, along with brainstorming ways to further promote interprofessional conversations.

## Discussion

This student-led IPE pilot program aimed to enhance students’ understanding of interprofessional collaboration in the healthcare setting. Sessions were designed to (1) address a lack of knowledge in the roles and responsibilities of the members of the healthcare team and (2) develop the participants’ confidence in their ability to effectively work within a diverse healthcare team. An emphasis was placed on navigating instances of miscommunication and bullying. Feedback from each session supported our theory that students of various healthcare professions can benefit from early exposure to interprofessional collaboration and communication, as evidenced by increased levels of confidence, self-perceived knowledge, and overall positive feedback after each session across students in every program. The resulting increased confidence in students’ ability to collaborate with other healthcare professionals after attending the sessions is consistent with other similar interprofessional lecture series [16,18-20].

The effectiveness of the seminar series is rooted in the active engagement of learners and the incorporation of discussions between peers and faculty. IPE offerings should include these elements to optimize student learning, and doing so is supported by social constructivist learning theory [21]. By having learners from various health professions and training programs interact with one another, the participants can operate within their zone of proximal development to gain a new understanding of the roles and responsibilities of other health professionals. Involving different faculty members from each profession over the seminar series exposed students to a wide range of perspectives and experiences, bringing to life the challenges of working on professionally diverse teams. Providing students the opportunity to learn about other healthcare professions from other healthcare professionals and trainees, especially as they change and develop over time, is a valuable opportunity in undergraduate medical education.

Limitations of this pilot study include a lack of a control group, self-reported data with potential response bias, no previous cohorts with which to compare these data, and no follow-up of the clinical utility of participating in these sessions. Further studies would be necessary to determine if there is a significant benefit for attendees of these sessions in their future professional collaborative capabilities and if there is any impact on the quality of patient care.

As a pilot program, opportunities for improvement were identified in the planning of the seminar series and evaluation methods. In the future, we plan to replicate this seminar series with minor changes to improve interprofessional discussion. For example, in session 3, we did not assign small groups in a manner that ensured diverse representation in the form of a student from each participating program. We also plan to incorporate a more longitudinal series for students from all programs across an entire school year, rather than three months, to learn within the same cohort over time.

## Conclusions

As healthcare roles and responsibilities evolve, it becomes increasingly important to educate healthcare profession students on the educational background and capabilities of their future colleagues, as well as how to effectively collaborate to benefit patient care. These sessions provided a unique opportunity for students in different healthcare programs to meet one another, gain exposure to realistic scenarios and actual experiences from panelists, and learn about the roles of their future colleagues. The design and implementation of this interprofessional series, being led by students from a variety of academic programs, coupled with the involvement of faculty from a broad range of health profession backgrounds, resulted in a diverse representation of presenters and attendees. Future endeavors include recreating the seminar series and gathering additional data, as well as studying past participants in the clinical setting to assess the impact of experiences gained through these sessions.

## References

[1] Rowan BL, Anjara S, De Brún A, MacDonald S, Kearns EC, Marnane M, McAuliffe E (2022). The impact of huddles on a multidisciplinary healthcare teams' work engagement, teamwork and job satisfaction: a systematic review. J Eval Clin Pract.

[2] Shaw M, Pelecanos AM, Mudge AM (2020). Evaluation of internal medicine physician or multidisciplinary team comanagement of surgical patients and clinical outcomes: a systematic review and meta-analysis. JAMA Netw Open.

[3] Guraya SY, Barr H (2018). The effectiveness of interprofessional education in healthcare: a systematic review and meta-analysis. Kaohsiung J Med Sci.

[4] Stenberg K, Mills R, Kalia I, Schwartz L (2025). Genetic counselors' professional identity in North America: a scoping review. J Genet Couns.

[5] Johnson AW, Potthoff SJ, Carranza L, Swenson HM, Platt CR, Rathbun JR (2006). CLARION: a novel interprofessional approach to health care education. Acad Med.

[6] Salvatori PS, Berry SC, Eva KW (2007). Implementation and evaluation of an interprofessional education initiative for students in the health professions. Learn Health Soc Care.

[7] Honan L, Fahs DB, Talwalkar JS, Kayingo G (2015). Interprofessional learning: perceptions of first year health students. J Nurs Educ Pract.

[8] (2025). Timeline of the genetic counseling profession. https://www.nsgc.org/About/About-NSGC/Timeline.

[9] Hovorka CF, Shurr DG, Bozik DS (2002). The concept of an entry-level interdisciplinary graduate degree preparing orthotists for the new millennium part 1: history of orthotic and prosthetic education. J Pros Ortho.

[10] Hammoud MM, Appelbaum NP, Wallach PM (2021). Incidence of resident mistreatment in the learning environment across three institutions. Med Teach.

[11] Physician Assistant Education Association (2020). Physician Assistant Education Association: By the numbers: student report 4: data from the 2019 matriculating student and end of program surveys. By the Numbers: Student Report 4: Data From the 2019 Matriculating Student and End of Program Surveys.

[12] Elisha S, Rutledge DN (2011). Clinical education experiences: perceptions of student registered nurse anesthetists. AANA J.

[13] Reeves S, Perrier L, Goldman J, Freeth D, Zwarenstein M (2013). Interprofessional education: effects on professional practice and healthcare outcomes (update). Cochrane Database Syst Rev.

[14] van Diggele C, Roberts C, Burgess A, Mellis C (2020). Interprofessional education: tips for design and implementation. BMC Med Educ.

[15] Averbuch T, Eliya Y, Van Spall HG (2021). Systematic review of academic bullying in medical settings: dynamics and consequences. BMJ Open.

[16] Berger S, Mahler C, Krug K, Szecsenyi J, Schultz JH (2016). Evaluation of interprofessional education: lessons learned through the development and implementation of an interprofessional seminar on team communication for undergraduate health care students in Heidelberg - a project report. GMS J Med Educ.

[17] Kirkpatrick D, Kirkpatrick J (2006). Evaluating Training Programs: The Four Levels. Berrett-Koehler, Oakland.

[18] Gowda D, Curran T, Khedagi A (2019). Implementing an interprofessional narrative medicine program in academic clinics: feasibility and program evaluation. Perspect Med Educ.

[19] Sakai DH, Marshall S, Kasuya RT (2012). Medical school hotline: interprofessional education: future nurses and physicians learning together. Hawaii J Med Public Health.

[20] Klocko DJ, Hoggatt Krumwiede K, Olivares-Urueta M, Williamson JW (2012). Development, implementation, and short-term effectiveness of an interprofessional education course in a school of health professions. J Allied Health.

[21] Vygotsky LS, Cole M (1978). Mind in Society: Development of Higher Psychological Processes.

